# Supplementary material for: Predictive Markers of Treatment Response to Neoadjuvant Systemic Therapy with Dual HER2-Blockade
Source: Cancers (Basel). 2024 Feb 19;16(4):842. doi: 10.3390/cancers16040842 (PMC10886516; doi:10.3390/cancers16040842)
Supplement: Supplementary file 1 [file cancers-16-00842-s001.zip › cancers-2849453-supplementary.pdf]

Table S1. Baseline characteristics according to tumor-infiltrating lymphocyte levels.

Variables	low TIL levels	high TIL levels	Total	*P* value
Age				0.653
< 50	90 (48.9)	56 (46.3)	146 (47.9)	
≥ 50	94 (51.1)	65 (53.7)	159 (52.1)	
HG*				0.001
1 or 2	161 (87.5)	86 (72.9)	247 (81.8)	
3	23 (12.5)	32 (27.1)	55 (18.2)	
HR				0.011
positive	99 (53.8)	47 (38.8)	146 (47.9)	
negative	85 (46.2)	74 (61.2)	159 (52.1)	
ER expression				0.034
≥ 10%	85 (46.2)	39 (32.2)	124 (40.7)	
1-9%	14 (7.6)	8 (6.6)	22 (7.2)	
0	85 (46.2)	74 (61.2)	159 (52.1)	
PR expression				0.087
≥ 10%	43 (23.4)	18 (14.9)	61 (20.0)	
1-9%	18 (9.8)	8 (6.6)	26 (8.5)	
0	123 (66.8)	95 (78.5)	218 (71.5)	
HER2				0.870
IHC 2+	41 (61.2)	143 (60.1)	184 (60.3)	
IHC 3+	26 (38.8)	95 (39.9)	121 (39.7)	
Clinical T stage				0.026
1	4 (2.2)	4 (3.3)	8 (2.6)	
2	97 (52.7)	81 (66.9)	178 (58.4)	
≥ 3	83 (45.1)	36 (29.8)	119 (39.0)	
Clinical nodal status				0.207
negative	43 (23.4)	21 (17.4)	64 (21.0)	
positive	141 (76.6)	100 (82.6)	241 (79.0)	
Ki-67 (%)*, median (IQR)	30 (20-60)	45 (20-70)	30 (20-60)	0.200

*Missing value

TIL = tumor-infiltrating lymphocyte, HG = histologic grade, HER2 = human-epidermal growth receptor factor 2, HR = hormone receptor, ER = estrogen receptor, PR = progesterone receptor, IHC = immunohistochemistry.
